# Anticonvulsant and neuroprotective effects of *Pimpinella anisum* in rat brain

**DOI:** 10.1186/1472-6882-12-76

**Published:** 2012-06-18

**Authors:** Fariba Karimzadeh, Mahmoud Hosseini, Diana Mangeng, Hassan Alavi, Gholam Reza Hassanzadeh, Mohamad Bayat, Maryam Jafarian, Hadi Kazemi, Ali Gorji

**Affiliations:** 1 Shefa Neuroscience Center, Tehran, Iran; 2 Mashhad Neuroscience Center, University of Medical Sciences, Mashhad, Iran; 3 Department of Physiology, Mashhad University of Medical Sciences, Mashhad, Iran; 4 Institut für Physiologie I, Westfalische Wilhelms-University Münster, Münster, Germany; 5 Department of Anatomy, Tehran University of Medical Sciences, Tehran, Iran; 6 Department of Pediatric, Shahed University, Tehran, Iran; 7 Razavi Neuroscience Center, Mashhad, Iran

**Keywords:** Cephalic pain, Stroke, Brain ischemia, Medical plants, Traditional therapy

## Abstract

**Background:**

Essential oil of *Pimpinella anisum L. Apiaceae* (anise oil) has been widely used in traditional Persian medicine to treat a variety of diseases, including some neurological disorders. This study was aimed to test the possible anti-seizure and anti-hypoxia effects of anise oil.

**Methods:**

The effects of different concentrations of anise oil were tested on seizure attacks induced by pentylenetetrazol (PTZ) injection and neuronal hypoxia induced by oxygen withdrawal as well as on production of dark neurons and induction of long-term potentiation (LTP) in *in vivo* and *in vitro* experimental models of rat brain.

**Results:**

*Anise oil* significantly prolonged the latency of seizure attacks and reduced the amplitude and duration of epileptiform burst discharges induced by injection of intraperitoneal PTZ. In addition, anise oil significantly inhibited production of dark neurons in different regions of the brain in epileptic rats. *Anise oil* also significantly enhanced the duration of the appearance of anoxic terminal negativity induced by oxygen withdrawal and inhibited induction of LTP in hippocampal slices.

**Conclusions:**

Our data indicate the anticonvulsant and neuroprotective effects of anise oil, likely via inhibition of synaptic plasticity. Further evaluation of anise oil to use in the treatment of neurological disorders is suggested.

## Background

Traditional knowledge, obtained from ancient and medieval medical literatures or from folkloric medicine, can play an important role in developing new drugs [1]. The investigation for new medicaments obtained from plants has led to the discovery of some clinically useful drugs that, during the past two centuries, have played a major role in the treatment of human diseases and contributed to our understanding of the pathophysiological mechanisms [2].

*Pimpinella anisum L.*, a flowering plant in the family Apiaceae, enjoys considerable reputation as a drug in medieval literatures. *Pimpinella anisum* is native to the eastern Mediterranean region and southwest Asia. Variations in the essential oil composition of *Pimpinella anisum* obtained from different geographical areas were reported [3]. The major constituents of *Pimpinella anisum* are trans-anethole, estragole, and panisaldehyde [3,4]. Ingestion of as little as 1–5 ml of *Pimpinella anisum* seed oil (anise oil) can result in nausea, vomiting, seizures, and pulmonary edema. The LD50 value per kg body weight for anise oil is 2.7 g [5].

Essential oil of *Pimpinella anisum* (anise oil) is used today as an ingredient in cough medicine and reported to have diuretic and diaphoretic properties [6]. *Pimpinella anisum* has been also used as carminative, antiseptic, antispasmodic, expectorant, stimulant and stomachic medicaments. In addition, it has been used to promote lactation in nursing mothers and as a medicine against stress, bronchitis, indigestion and lice. In Persian medieval medicine, the plant and especially its fruit essential oil have been used for treatment of some neurological disease, including seizures and epilepsy [7]. Based on Persian folk medicine, anise oil is also believed to have protective effects against the development of cerebrovascular diseases [8].

Only a few studies pointed to the possible effects of *Pimpinella anisum* on neuronal activity. The aqueous extract of leaves and stems of anise oil has been reported to postpone the onset of picrotoxin-induced seizure attacks in mice [9]. A study investigated anti-epileptic effects of anise oil against seizures induced by pentylenetetrazol (PTZ) or maximal electroshock in male mice revealed suppressive effects of the substance on tonic convulsions induced by both models. Application of intraperitoneal anise oil diluted in sesame oil at concentration range of 0.25-1 ml/kg also enhanced the threshold of PTZ-induced clonic convulsions in mice [10]. Pentobarbital–induced sleeping time was prolonged by intraperitoneal administration of anise oil (50 mg/kg) to mice [5]. The aim of the present study was to examine the probable cerebroprotective and anticonvulsant effects of anise oil. Different concentrations of the compound were examined by *in vitro* and *in vivo* models of hypoxia and epilepsy in rats.

## Methods

### In vivo studies

#### Electroencephalogram (EEG) recordings and PTZ-injection

Adult male Wistar rats, weighing 250–300 g (n = 32), were housed under a constant temperature (22 °C) and illuminated 7:00 a.m. to 7:00 p.m., with food pellets and water available ad libitum.

Recording electrodes were stereotaxically implanted on the dura mater of the left and right somatosensory cortex under intraperitoneal urethane anesthesia (1.2 g/kg). EEG was recorded by silver electrodes connected to an amplifier (EXT-02 F, NPI, Germany) and stored by a digital oscilloscope. EEG recordings were performed for 30 min before (control) and one hour after PTZ (120 mg/kg, i.p.; Sigma, dissolved in saline) injection [11]. PTZ was administered intraperitoneally*.* Latency, duration, amplitude, and frequency of spikes were calculated using AxoScope software.

Rats were randomly divided in four groups as follows:

1- Control group: the animals of this group didn’t receive any surgery procedure or EEG recording. The brains were removed and histological studies were carried out (n = 8).

2- Sham Group: the animals of this group underwent the surgery procedure. The electrodes were implanted on the dura mater and EEG was recorded. Finally, the brains were removed and histological studies were carried out (n = 8).

3- PTZ group: the electrodes were implanted the same as group 2. EEG was recorded before and after PTZ injection. Finally, the brains were removed for histological studies (n = 8).

4- Treatment group: the surgery and electrode implantation was the same as the PTZ group. The animals were treated by 1 (Pi 1), 2 (Pi 2) or 3 (Pi 3) ml/kg of anise oil (intraperitoneal injection*;* 9 mg/ml)*,* 30 min before PTZ injection (n = 8). Anise oil was dissolved in ethanol. The final concentration of ethanol was less than 0.1%. All solutions used in control periods contained the same concentration of ethanol.

#### Histological studies

One hour after finishing experimental protocols (see above), all rats from different groups (n = 32) were given a high dose of urethane and transcardially perfused with 100 ml of saline followed by 100 ml fixative solution (glutaraldehyde 1.25 % and paraformaldehyde %1 in 0.2 M buffer phosphate at pH = 7.4; [12,13]). After perfusion, all rats were decapitated and the brains removed. The brains were kept in 4 % PFA for at least 1 week and were then processed for histological studies as follows. Three series of 10-μm thick coronal sections were cut every 100-μm from 2.3 to 4.3 mm posterior to the bregma [14,15]. Sections were stained with hematoxylin and eosin, crystal violet, and toluidine blue. Slides were examined with light microscope and digital photographs were taken from hippocampal CA1 and CA3 areas of both hemispheres. For quantitative analysis of dark neurons, the physical dissector method was used [13,15]. Ten pairs of sections, with 8 mm distance, were collected from each brain. The first section of each pair was designated as the reference and the second one was used for comparison. On each pair of sections, at least 10 microscopic fields were selected by uniform systematic-random sampling in every area of interest. Using the unbiased frame and physical dissector counting rule, the counting of dark neurons in each field was carried out.

### In vitro studies

Adult male rats (250–300 g; n = 24) were decapitated under deep methohexital anaesthesia and the brains were rapidly removed to cold (4 °C) artificial cerebrospinal fluid (ACSF). The medial hippocampi were dissected and cut into slices of 500 μm thickness. Slices were stored at 28 °C in ACSF, which contained (in mM) NaCl, 124; KCl, 4; CaCl2, 1.0; NaH2PO4, 1.24; MgSO4, 1.3; NaHCO3, 26; glucose, 10 (pH 7.4), carboxygenated with 95 % O2 and 5 % CO2 for 1 hour. After 30 min incubation, CaCl2 was elevated to 2.0 mM. Slices were individually transferred to an interphase recording chamber, placed on a transparent membrane, illuminated from below and continuously perfused (1.5–2 ml/min) with carboxygenated ACSF at 32 °C. A warmed, humified 95 % O2 and 5 % CO2 gas mixture was directed over the surface of the slices.

#### Electrophysiological recordings

Direct current (DC) extracellular field potentials and evoked potentials (EPs) were recorded with glass microelectrodes (150 mM NaCl; 2–10 MΩ) connected to the amplifier (Homemade extracellular amplifier, Münster, Germany) by an Ag/AgCl–KCl bridge. Extracellular glass micropipettes were placed into the stratum pyramidale of the CA1 region under visual control using optical microscope. These microelectrodes were used to record field excitatory postsynaptic potential (fEPSP) in response to stimuli (100–150 mA, 0.5 msec pulses) from paired wire electrodes placed ~1 mm from the recording site. Field potentials were traced by an ink-writer (Rikadenki, Freiburg, Germany) and recorded by a digital oscilloscope (Nicolet Instrument Technologies, USA). The ink-writer traced the sustained potential shifts and the oscilloscope recorded EPs from the same microelectrode.

EPs were defined as the population spike following an electrical stimulation and riding on fEPSP. Amplitudes were defined as the difference between positive fEPSP peaks and a negative population spike peak. In all experiments, amplitudes of EPs were stable for 30 min prior to initiation of hypoxic conditions.

#### Induction of Hypoxia

Hypoxic conditions were induced only one time in each slice by replacing O_2_ against N_2_ in the fluid and gaseous phase with the perfusion of the slice being continued. As a consequence, a negative shift of DC potential (anoxic terminal negativity; ATN) developed. Hypoxic conditions were terminated as soon as ATN wave had reached a plateau. EPs amplitudes were measured at least 30 min before and after hypoxia and compared to each other. Latency of ATN was determined from the beginning of N_2_ application to the point at which 10 % of the maximum ATN amplitude had been reached. ATN amplitude was measured from baseline to peak. One slice was used for each experiment.

#### Long-term potentiation (LTP)

Single electrical stimulation (0.05–0.1 Hz) was applied through a bipolar platinum electrode attached to the Schaffer collaterals of hippocampal slices. fEPSP were elicited by adjusting the intensity of stimulation to 50 % of that at which population spikes after fEPSP began to appear in the hippocampus. In LTP experiments, the hippocampus was sequentially stimulated once every minute. Tetanic stimulations were applied when the amplitude of fEPSP remained stable with a maximum difference of 10 % for at least 30 min. Ten trains of four pulses at 100 Hz were delivered 200 ms apart to the Schaffer collaterals of hippocampal slices. LTP was operationally defined as the mean change in fEPSP slope amplitude (1 ms after the onset of response) for five intensity stimuli given beginning 30 min after tetanic stimulation compared with the mean response to five test pulses given immediately before the stimulation. Tetanic stimulation was applied 60 min after application of anise oil.

Essential oil of anise oil purchased from Caelo (Caesar & Loretz GmbH, Caelo, Hilden, Germany). *N*-methyl-*D*-aspartic acid (NMDA), DL*-*2*-*amino*-*5*-*phosphonovaleric acid (APV), 6-cyano-7-nitroquinoxaline-2,3-dione (CNQX) and PTZ purchased from Sigma (St. Louis, MO, USA). Anise oil was characterized mainly by *trans*-anethole (89.1 %), estragol (3.6 %), linalool (1.1 %), *α*-terpineol (0.2 %) and *cis*-anethole (0.2 %) [16]. The investigations were approved by the local ethics committee (Ethikkommission der Arztekammer Westfalen-Lippe und der Medizinischen Fakultut der Universitat Münster and Shefa Ethics Committee).

### Statistical analysis

All data are given as mean ± S.E.M. The data were statistically analyzed using the Mann–Whitney rank sum test. Multiple comparisons were performed by analysis of variance test (ANOVA) for repeated measures followed by a Duncan’s test. Significance was established when the probability values were less than or equal to 0.05.

## Results

### The effect of anise oil on PTZ-induced seizures

**Figure 1:**
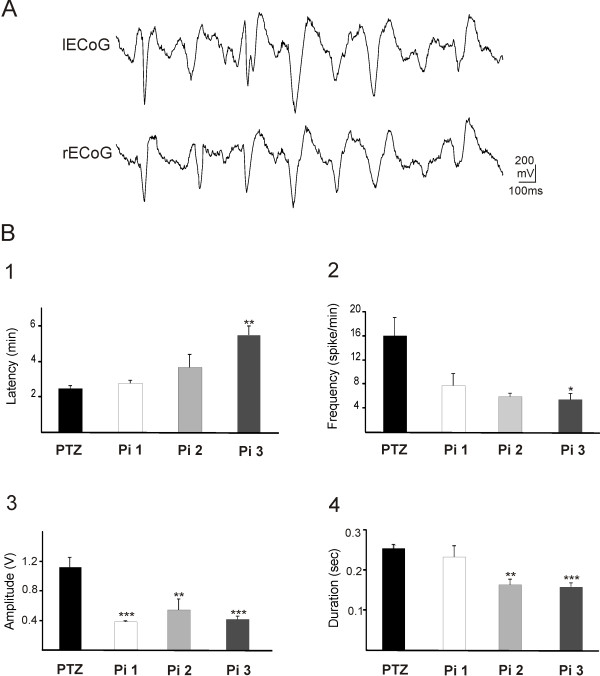
**The effect of anise oil on epileptic activities induced by injection of pentylenetetrazol (PTZ) in rats.** (**A**) Sample of epileptiform burst discharges recorded by electrocorticogram (ECoG) from left (l) and right (r) hemispheres. (**B**) The effect of *Pimpinella* at concentrations of 1 (Pi1), 2 (Pi2), and 3 (Pi3) ml/kg on the latency (1), frequency (2), amplitude (3), and duration (4) of epileptiform activities induced by PTZ injection in rat *in vivo* model. The latency time of spikes in Pi3 group was significantly longer than PTZ group (*p* < 0.01). The amplitude of spikes in Pi1, Pi2, Pi3 groups was significantly lower than PTZ group (**p < 0.01and ***p < 0.001). Duration of spikes in groups Pi2, Pi3 was also significantly shorter than PTZ group (**p < 0.01and*** *p* < 0.001). The frequency of burst discharges in Pi3 group was significantly lower than PTZ group (*p = 0.035).

PTZ at concentration of 120 mg/kg induced clonic seizures in all anesthetized rats. The onset of seizures occurred after 2.4 ± 0.18 min in PTZ group. A seizure induced by PTZ typically started with hind limb kicks, followed by generalized tonic and clonic convulsions of four limbs while the rats laying down. EEG was monitored to confirm the seizure occurrence. Epileptiform burst discharges were observed during seizure attacks (Figure 1, A). The frequency, amplitude and duration of these potentials in PTZ group were 16 ± 2.9 / min, 1100 ± 10 μV and 250 ± 10 ms, respectively.

Administration of anise oil at concentrations of 1 or 2 ml/Kg did not change the latency of seizure attacks after PTZ injection. However, anise oil at concentration of 3 ml/Kg significantly prolonged the appearance of seizures to 5.43 ± 0.49 min after PTZ injection (*p* < 0.01; Figure 1, B1). Anise oil at all different concentrations significantly decreased the frequency, amplitude, and duration of epileptiform burst discharges induced by PTZ injection. Anise oil at concentrations of 1, 2, or 3 ml/Kg inhibited the repetition rate of epileptic discharges to 6 ± 0.57, 7.66 ± 2.02, and 5.33 ± 2.9 per minute, respectively (*p* = 0.035; Figure 1, B2). The amplitude of the epileptiform potentials in all three groups of animals treated with 1, 2, or 3 ml/Kg of anise oil was reduced to 501 ± 14, 298 ± 11, and 318 ± 30 μV, respectively (*p* < 0.001; Figure 1, B3). The mean duration of PTZ-induced burst discharges was also significantly reduced to 230 ± 19, 160 ± 13, and 150 ± 12 ms after administration of 1, 2, or 3 ml/kg of anise oil, respectively (*p* < 0.001; Figure 1, B4).

### The effect of anise oil on production of dark neurons

**Figure 2:**
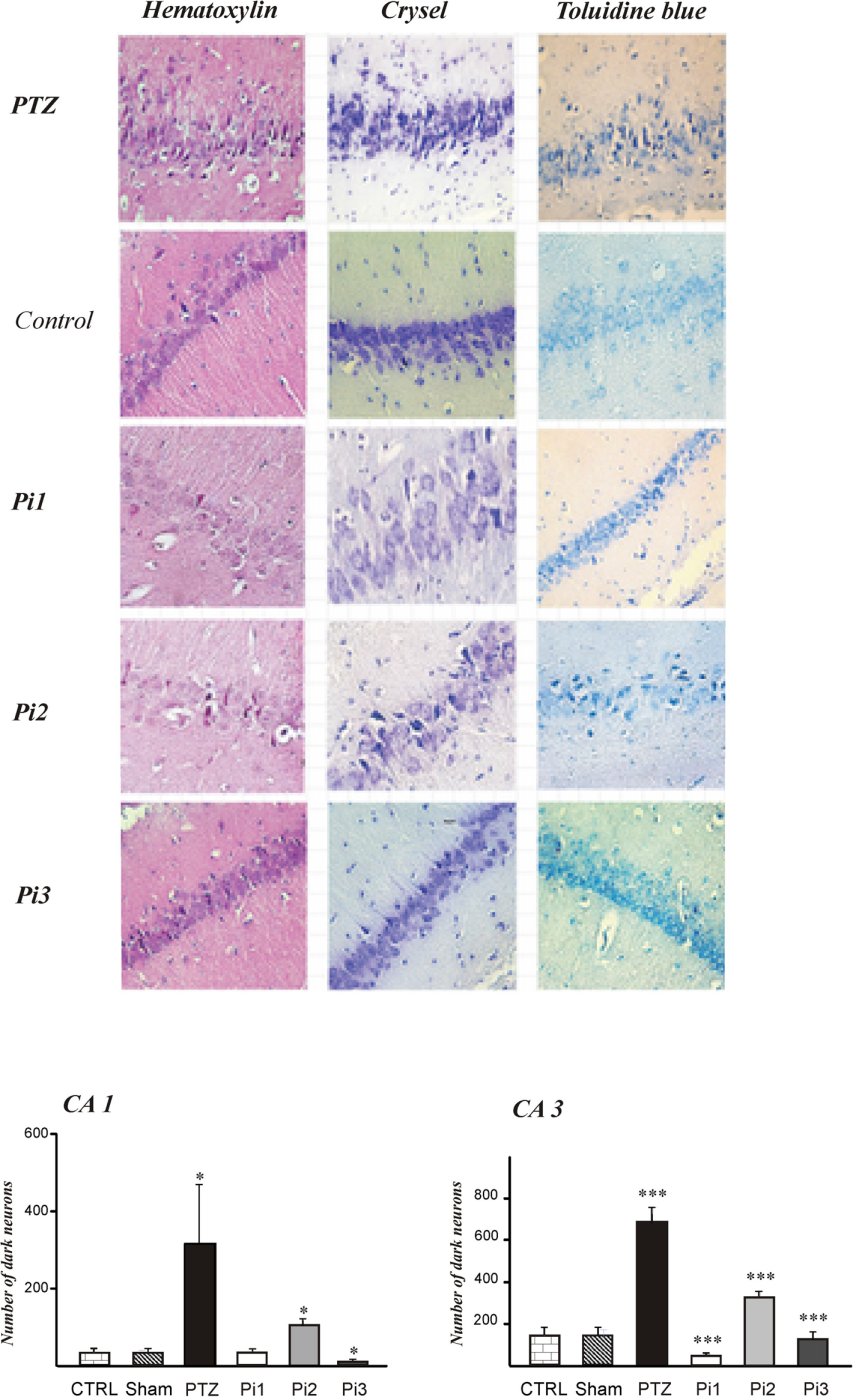
**The inhibitory effect of anise oil on production of dark neurons.** Anise oil at concentrations of 1 (Pi1), 2 (Pi2), and 3 (Pi3) ml/kg inhibited production of dark neurons in the hippocampal CA1 and CA3 areas in rats after induction of seizure attacks by intraperitoneal pentylenetetrazol (PTZ) injection. (A) Light-microscopic appearance of hematoxilin and eosin (left), crystal violet (middle), and toluidine blue (right) stained dark neurons in 10-μm sections of the CA3 in rats. Dark neurons number in both CA1 and CA3 regions of PTZ group was significantly more than sham and control groups, while anise oil reduced significantly the number of dark neurons in both areas in PTZ group (* p < 0. 01 and ***p < 0.001).

Dark neurons were identified by the neuronal shrinkage, cytoplasmic esoinophilia, nuclear pyknosis, and surrounding spongiosis. Density of dark neurons in the hippocampal CA1 and CA3 areas was significantly decreased after administration of anise oil in comparison to control, sham and PTZ groups. The mean number of dark neurons was 24 ± 4 in control rats, 33 ± 5 in sham rats, and 316 ± 15 in PTZ groups in hippocampal CA1area. Injection of PTZ enhanced the density of dark neurons in CA1 area of the hippocampus (*p* < 0.05). Administration of anise oil at concentration of 1 ml/Kg did not change the density of dark neurons induced by PTZ injection in this brain area (104 ± 18). However, injection of 2 and 3 ml/Kg of anise oil significantly prevented production of dark neurons by PTZ injection in CA1 region and reduced the mean number of dark neurons to 25 ± 9, and 10 ± 7, respectively (*p* < 0.05; Figure 2).

The mean number of dark cells in hippocampal CA3 area in control, sham, and PTZ groups was 112 ± 11, 140 ± 36, and 688 ± 77, respectively. The number of dark neurons in CA3 region of PTZ treated rats was significantly higher than sham and control groups. Anise oil prevented induction of dark neurons by PTZ injection. Administration of anise oil at concentrations of 1, 2, and 3 ml/Kg before injection of PTZ decreased the mean number of dark neurons in CA3 area to 315 ± 45, 128 ± 36, and 48 ± 36, respectively (*p* < 0.001; Figure 2).

### The effect of anise oil on induction of hypoxia

**Figure 3:**
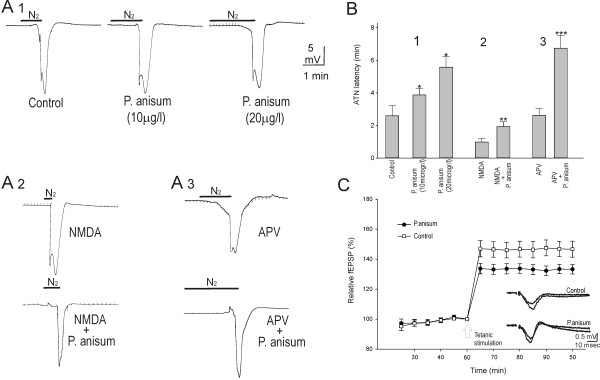
**The effect of anise oil on hypoxic events and synaptic plasticity**. Anise oil extended the latency of the induction of anoxic terminal negativity (ATN) by hypoxia (deprivation of O2) and inhibited induction of long-term potentiation (LTP) in rat hippocampal slices. (**A**) Recordings of ATN of the DC-potential and of evoked potentials in control (**A1**), with pre-treatment of anise oil (**A2**; 20 μg/l), with pretreatment with APV (**A3**; 10 μmol/l), and pre-treatment with APV and anise oil (**A4**) in CA1 areas of the hippocampus. (**B**) Statistical evaluation of the latency of the appearance of ATN in control as well as pre-treatment with anise oil at 10 and 20 μg/l (**B1**; * indicate *p* = 0.03, ANOVA test), pre-treatment with NMDA as well as NMDA and anise oil (**B2**; ** indicate P = 0.007, Mann-Whitney rank sum test), pre-treatment with APV (**B3**; 10 μmol/l), and pre-treatment with APV and anise oil (**B4**; ** indicate *p* = 0.04, Mann-Whitney rank sum test). Anis oil significantly extended the latency of the induction of ATN, possibly by inhibition of glutamate NMDA sub-receptors. (**C**) Tetanic stimulation produces a rapid and stable potentiation in the amplitude of the evoked field potentials, calculated as a percentage of baseline mean response amplitude. (**C1**) Open triangle and solid circles show the evoked fEPSP after application of anise oil and control, respectively. Arrow shows the time of tetanic stimulation, 60 min after application of anise oil (20 μg/l) or ACSF (control). The time points given refer to substance application. Application of anise oil significantly inhibited LTP of the evoked field potentials (Mann-Whitney rank sum test, *p* = 0.006), calculated as a percentage of baseline mean response amplitude. (**C2**) Representative examples of the evoked field potentials before and after titanic stimulation in control and anise oil -treated slices.

In control group, with hypoxic condition (deprivation of oxygen), ATN occurred in all slices after 2.59 ± 0.6 min with the amplitude of 15.9 ± 2.5 mV (n = 6). The neuroprotective effect of three different concentrations of anise oil (1, 10, and 20 μg/l) was tested in the hippocampus. To reach the possible maximum effect, anise oil was applied for 60 min before induction of hypoxia. Administration of anise oil at concentration of 1 μg/l, one hour before deprivation of oxygen, neither extended ATN latency nor changed the amplitude and duration of ATN. However, administration of anise oil at concentrations of 10 and 20 μg/l significantly extended ATN latency to 3.9 ± 0.4 and 5.6 ± 0.6 min, respectively (P = 0.03, ANOVA; Figure 3, A1 and B). Anise oil at 10 and 20 μg /l did not affect the amplitude and the duration of ATN. Recovery of evoked potential to pre-hypoxia level was observed 5–10 minutes after termination of hypoxic condition in all slices.

Glutamate receptors play a key role in hypoxic brain damage, and that drugs which decrease the accumulation of glutamate or block its synaptic effects on NMDA and non-NMDA receptor sites may be a rational therapy for stroke (Hossmann, 1994). Addition of NMDA (10 μM) to the bath solution one hour before induction of hypoxia significantly shortened ATN latency compare to control slices (0.97 ± 0.3 min, P = 0.039). Addition of anise oil at concentrations of 20 μg/l and NMDA (10 μM) to the bath medium extended ATN latency to 1.94 ± 0.3 min (P = 0.007; Figure 3, A2 and B). Administration of NMDA alone or with anise oil did not affect the amplitude of ATN. Furthermore, 60 min administration of APV (NMDA antagonist; 10 μM) had no effect on the latency and amplitude of ATN (2.63 ± 0.4 min and 13.8 ± 2.4 mV). However, addition of APV (10 μM) and anise oil at concentrations of 20 μg/l to the superfuste significantly extended the latency of ATN (6.8 ± 0.8 min) in comparison to the slices washed by anise oil alone (P = 0.04; Figure 3, A3 and B). Combination of APV and anise oil did not affect the amplitude of ATN. Addition CNQX (10 μM) or CNQX and anise oil did not change the characteristic features of ATN compare to control condition and administration of anise oil alone, respectively.

### The effect of anise oil on induction of LTP

A conditioning tetanic stimulation was delivered to the Schaffer collaterals of hippocampal tissues followed by pulses with stimulation parameters identical to controls 60 min after application of anise oil or ACSF (control group) in the hippocampus. The amplitudes of fEPSP were stable for at least 30 min before induction of tetanic stimulation (less than 10 % variation). Tetanic stimulation in control slices produced a rapid, stable and lasting enhancement of the amplitude of fEPSP in all tested preparations (n = 7, 147.3 ± 5 % control). The potentiation rose within 2–3 min and stabilized within 4–6 min after the stimulations. Addition of anise oil (20 μg/l; n = 8) to the bath medium 60 min before tetanic stimulation significantly decreased LTP induction in all tested slices (134 ± 4 % control, Mann–Whitney rank sum test; P = 0.006; Figure 3, C).

## Discussions

The present data indicate the significant effects of anise oil to protect neurons against hypoxia as well as its anticonvulsant properties in *in vitro* and *in vivo* animal brain models. Data indicated that pretreatment with anise oil increased the latency and reduced the amplitude and duration of epileptiform burst discharges induced by PTZ injection. Anise oil also extended ATN latency, an effect enhanced by blocking of NMDA receptors. Furthermore, our findings revealed a remarkable reduction of dark neuron production and LTP induction in the hippocampus of rats pre-treated with anise oil.

In line with our results, anti-epileptic effects of anise oil were reported in different animal epilepsy models. Pourgholami et al. [10] showed that essential oil of anise exerted anticonvulsant effects induced by PTZ or maximal electroshock in male mice. Anticonvulsant effect of anise oil was also reported in picrotoxin model of epilepsy in mice [9]. In contrary to these data, it is reported that application of anise oil (0.01 or 0.05 %) alone or in the presence of PTZ elicited neuronal burst discharges or enhanced the burst firing and the steepness of the paroxysmal shift induced in an *in vitro* model of epilepsy in snail. In this model, anise oil also induced irregularity in firings of tonic discharges and decreased the after-hyperpolarizing potential in intact cells [17]. This discrepancy may be related to the different epilepsy models used in these studies. Some other anticonvulsant substances such as benzodiazepines elicit epileptiform burst discharges in snail neurons [18].

Anticonvulsant effects of anise oil may be due to activation of GABA_A_ receptors [19]. It has been shown that anise oil exerts its effect on opioid receptors via activation of GABA_A_ receptors in mice. In addition, it has been revealed that anise oil enhances the activity of the Na^+^-K^+^ ATPase [20]. The Na^+^-K^+^ pumps play an important role in the regulation of neuronal excitability. Disruption of the pump activity is suggested as a mechanism in the development of epileptiform burst discharges. Na^+^-K^+^ pump inhibition altered both GABA_A_ and GABA_B_ components of inhibitory post-synaptic potentials [21].

In this study, the main constituents of anise oil are *trans*-anethole (89.1 %), estragol (3.6 %), linalool (1.1 %), *α*-terpineol (0.2 %) and *cis*-anethole (0.2 %) [16]. All these active agents may be responsible for the effects observed in this study. The main component of anise oil is anethole (1-(4-Methoxyphenyl)-1-propen) [16]. Anethole is largely used as a substrate for the synthesis of various substances of neuro-pharmaceutical interest such as chloral, an anticonvulsant, and amphetamine [22]. Anethole also is a substrate for the synthesis of pentobarbital, a potent anticonvulsant drug [23]. Pentobarbital has been also shown to decrease cerebral metabolism as well as infarct volume and maintain the cerebral energy state during ischemia [24]. The present study investigated the neuroprotective effects of anise oil in hippocampal CA1 area. Pyramidal neurons in the CA1 region of the hippocampus are highly vulnerable to damage from hypoxic conditions, whereas cells in the CA3 region and the dentate gyrus are more resistant [25]. The key role of glutamate accumulation and activation of NMDA receptors in the pathophysiology of cerebral ischemia is well established [26]. Ischemia or hypoxia promotes enhanced release of the glutamate in the brain. Excessive stimulation of NMDA subtype receptors by glutamate results in a massive depolarization concomitant with excessive amounts of Ca^2+^ accumulating within the neuron. This may subsequently lead to cell death. Antagonists acting within the ion channel of the NMDA receptor have been shown to protect susceptible neurons against the deleterious consequences of ischemia/anoxia [27]. In the present study, addition of APV at low concentration enhanced neuroprotective effect of anise oil.

Furthermore, our data indicate that anise oil modulates synaptic plasticity in the hippocampus. Induction of LTP was inhibited by application of anise oil. LTP is a phenomenon in which a constant presynaptic high stimulation of excitatory amino acids results in a prolonged enhanced synaptic response. It is well established that NMDA receptors are a molecular detector of the coincidence of both the presynaptic release of glutamate and a postsynaptic depolarization at the origin of LTP induction [28]. It has been shown that a short period of hypoxia can induce a form of LTP of the pre-synaptic response at hippocampal CA1-CA3 synapses. This represents a pre-synaptic hyperexcitability of the afferent fibers following hypoxia, and may responsible for the excitotoxicity to the neurons induced by increases of glutamate release and the postsynaptic hypoxic LTP [29]. Inhibition of LTP as well as neuroprotective effects of anise oil is likely to be mediated by inhibition of NMDA receptors. In addition to inhibitory effect of anise oil on epileptiform burst discharges, the present data indicate neuroprotective effect of anise oil. In line with this, some antiepileptic drugs such as levetiracetam and lamotrigine have shown neuroprotective effects in animal models of injury. In such a case, the therapeutic value of these drugs would include not only control of seizure activity, but also the prevention of injury responses. Mechanisms of basic action of antiepileptic medicaments that are crucial to anticonvulsant efficacy have been considered as having a role in neuroprotection [30].

In the present study, anise oil extended ATN latency but did not affect the amplitude and the duration of ATN. In several studies different characteristic features of ATN were affected differently by pharmacological manipulation [31,32]. These studies indicate that the latency of ATN is the most important characteristic feature of ATN which may be affected by neuroprotective substances [33-35].

Dark neurons are reported in clinical and experimental neuropathology from living neuronal tissues [36]. Dark neurons were first observed to occur in neurosurgical biopsies, but were not seen at autopsy. Because of the appearance of dark neurons after mechanical trauma to the brain prior to fixation, a mechanical stress force was hypothesized to produce these neurons [14]. However, dark neurons also appear under conditions where no trauma or mechanical forces are applied to the neuronal tissues. Several studies revealed that trauma is only one of the many processes for experimentally producing dark neurons [14]. It is concluded that neuronal trauma is not a prerequisite for the production of dark neurons, despite the fact that dark neurons can be produced by mechanical injuries. In perfusion-fixed brain tissue, dark neurons have been shown in epilepsy, ischemia, hypoglycaemia, exposure to excitatory amino acids and mechanical neuronal trauma [37]. It has been suggested that release of excitatory neurotransmitters such as glutamate and aspartate as well as neuronal transmembrane ion flux may lead to tissue perturbation and production of dark neurons [36,38,39]. Excessive excitability, glutamate release and elevation of intracellular calcium due to seizure induced by PTZ may cause cell death [39,40]. In the present study, anise oil prevented production of dark neurons by inhibition of seizure attacks and, therefore, may act as a neuroprotective substance.

## Conclusions

The present study indicates neuroprotective and anticonvulsive effects of anise oil, possibly via inhibition of synaptic activities. Further studies are needed to evaluate the main constituent responsible of these effects as well as the exact mechanism of action.

## Abbreviations

ACSF, Artificial cerebrospinal fluid; APV, DL-2-amino-5-phosphonovaleric acid; ATN, Anoxic terminal negativity; CNQX, 6-cyano-7-nitroquinoxaline-2,3-dione; DC, Direct current; EEG, Electroencephalogram; EPs, Evoked potentials; FEPSP, field excitatory postsynaptic potential; LTP, long-term potentiation; NMDA, N-methyl-D-aspartic acid; PTZ, Pentylenetetrazol.

## Competing interests

The authors declare that they have no competing interests.

## Authors' contributions

FK, MH and DM performed the studies and analyzed the data. AH, GRH and MB evaluated the data and analysis. MJ and HK conceived the study together and helped draft the manuscript. AG: conceived of the project, wrote and revised the manuscript. All authors read and approved the final manuscript.

## Pre-publication history

The pre-publication history for this paper can be accessed here:

http://www.biomedcentral.com/1472-6882/12/76/prepub
